# Phenotypic Modulation of Biofilm Formation in a *Staphylococcus epidermidis* Orthopedic Clinical Isolate Grown Under Different Mechanical Stimuli: Contribution From a Combined Proteomic Study

**DOI:** 10.3389/fmicb.2020.565914

**Published:** 2020-09-08

**Authors:** Marta Bottagisio, Pietro Barbacini, Alessandro Bidossi, Enrica Torretta, Elinor deLancey-Pulcini, Cecilia Gelfi, Garth A. James, Arianna B. Lovati, Daniele Capitanio

**Affiliations:** ^1^IRCCS Istituto Ortopedico Galeazzi, Laboratory of Clinical Chemistry and Microbiology, Milan, Italy; ^2^Department of Biomedical Sciences for Health, University of Milan, Milan, Italy; ^3^IRCCS Istituto Ortopedico Galeazzi, Milan, Italy; ^4^Medical Biofilm Laboratory, Center for Biofilm Engineering, Montana State University, Bozeman, MT, United States; ^5^IRCCS Istituto Ortopedico Galeazzi, Cell and Tissue Engineering Laboratory, Milan, Italy

**Keywords:** proteomics, orthopedics, prosthetic joint infections, biofilm, extracellular polymeric substance, *Staphylococcus epidermidis*, coagulase-negative staphylococci

## Abstract

One of the major causes of prosthetic joint failure is infection. Recently, coagulase negative *Staphylococcus epidermidis* has been identified as an emergent, nosocomial pathogen involved in subclinical prosthetic joint infections (PJIs). The diagnosis of PJIs mediated by *S. epidermidis* is usually complex and difficult due to the absence of acute clinical signs derived from the host immune system response. Therefore, analysis of protein patterns in biofilm-producing *S. epidermidis* allows for the examination of the molecular basis of biofilm formation. Thus, in the present study, the proteome of a clinical isolate *S. epidermidis* was analyzed when cultured in its planktonic or sessile form to examine protein expression changes depending on culture conditions. After 24 h of culture, sessile bacteria exhibited increased gene expression for ribosomal activity and for production of proteins related to the initial attachment phase, involved in the capsular polysaccharide/adhesin, surface associated proteins and peptidoglycan biosynthesis. Likewise, planktonic *S. epidermidis* was able to aggregate after 24 h, synthesizing the accumulation associate protein and cell-wall molecules through the activation of the YycFG and ArlRS, two component regulatory pathways. Prolonged culture under vigorous agitation generated a stressful growing environment triggering aggregation in a biofilm-like matrix as a mechanism to survive harsh conditions. Further studies will be essential to support these findings in order to further delineate the complex mechanisms of biofilm formation of *S. epidermidis* and they could provide the groundwork for the development of new drugs against biofilm-related infections, as well as the identification of novel biomarkers of subclinical or chronic infections mediated by these emerging, low virulence pathogens.

## Introduction

Prosthetic joint infections (PJIs) are one of the main causes of joint prosthetic failure with an incidence of 2.5% of total joint arthroplasty associated with an increase of risk following revision surgery (10%) (Romano et al., 2016). These numbers not only reflect the economic impact on the U.S. Health Care System (>$50,000 per revision) but also a noticeable risk of mortality – up to 7% for 80-year-old patients (Lentino, 2003). Analysis of the explanted infected prostheses revealed that the most frequently recovered isolates are *Staphylococcus aureus*, *Staphylococcus epidermidis*, and *Pseudomonas aeruginosa* (Berbari et al., 1998; Lentino, 2003; Zimmerli et al., 2004).

*S. epidermidis*, a Gram-positive, coagulase-negative bacterial species, has recently emerged as a common cause of numerous nosocomial infections associated with medical devices, particularly in immunocompromised adults and infants (Ziebuhr et al., 2006). This is mainly due to the presence of *S. epidermidis* on healthy human skin where it lives as a commensal bacterium. *S. epidermidis* represents 90% of all the aerobic Firmicutes of the skin microbiota (Balato et al., 2019). Interestingly, the skin of healthy people is colonized by 10–24 different strains of *S. epidermidis* at any time and the host-bacteria bond is mutually beneficial since it impairs the attachment of more virulent bacteria (i.e., *S. aureus*) through microbial competition while conferring the optimal habitat for bacterial growth (Fey and Olson, 2010).

Depending on the biological context in which this low virulence bacterium grows, *S. epidermidis* can be either a commensal or an accidental pathogen (Ziebuhr et al., 2006; Otto, 2009). The triggering event is usually the presence of an indwelling device that favors bacterial attachment and the subsequent accumulation of bacteria enmeshed in an extracellular matrix, known as biofilm (Patel, 2005; Fey and Olson, 2010). Indeed, one of the major virulent determinants of *S. epidermidis* is the ability to regulate the expression of genes involved in biofilm formation (Fey and Olson, 2010). Within the biofilm, microorganisms establish organized hierarchies similar to that of multicellular organisms; cell-to-cell signaling regulates the expression of genes implicated in survival mechanisms, depending on environment influences (Patel, 2005). In addition, biofilm provides the cells protection from the host immune system, as well as from antimicrobial treatments, fostering the acquisition of resistance determinants and the development of multi drug-resistant bacteria (Lee et al., 2018). In this context, *S. epidermidis* can easily survive for many years inside the infected host causing a severe chronic infection barely distinguishable from an aseptic loosening of the orthopedic device (Donlan and Costerton, 2002). Hence, the diagnosis of PJIs mediated by *S. epidermidis* is complex and challenging due to the absence of clear clinical signs derived from the host immune system.

The availability of whole bacterial genome sequences in public databases allowed the study of the expression of the entire genome of a microorganism, launching the post-genomic era (Sauer, 2003). Thanks to proteomics, it is now possible to assay the expression of target genes and evaluate their modulation in accordance with different growth conditions (Zhang et al., 2013). Profiling gene expression patterns in biofilm-producing bacteria through “omics” sciences is important in order to determine the genetic basis of biofilm formation, and could provide the groundwork for the development of new drugs against biofilm-related infections, as well as the identification of novel biomarkers (Yao et al., 2005). Thus, aiming at studying the molecular mechanisms leading to the biofilm formation, in this study the proteome of a clinical isolate and a reference strain of *S. epidermidis* was analyzed by means of two-dimensional difference in gel electrophoresis (2D-DIGE) and label-free liquid chromatography and tandem mass spectrometry (LC-MS/MS). These proteomic analyses were carried out to describe how protein expression changes when two different bacterial strains belonging to the same species grown in a dynamic culture system while analyzing how the culture conditions modulate these differences.

## Materials and Methods

### Bacterial Culture Conditions

*Staphylococcus epidermidis* GOI1153754-03-14 (Bottagisio et al., 2017) and *S. epidermidis* ATCC 35984 (American Type Culture Collection) were both cultured in their planktonic (free-floating) and sessile (adherent, biofilm-forming) form. In particular, the planktonic culture was carried out by culturing 1.5 × 10^8^ CFU/ml of *S. epidermidis* in brain heart infusion (BHI, Becton Dickinson) broth under vigorous agitation at 200 rpm at 37°C in aerobic conditions for both 24 and 48 h. Differently, sessile bacteria were dynamically cultured on sandblasted titanium coupons in the drip flow reactor (DFR, Biosurface Technologies Corporation) (Goeres et al., 2009) following the same experimental time points. Briefly, after preconditioning the titanium coupons with BHI, 1 ml of bacterial suspension (1.5 × 10^8^ CFU/ml) was inoculated on each coupon placed in the DFR channels. After a 2-h incubation at 37°C, the peristaltic pump (Cole-Parmer Masterflex L/S) was activated to provide a continuous flow of medium in each channel of the bioreactor at a flow rate of 10 ml per hour for dynamic culture. After 24 and 48 h, each titanium coupon was washed three times with ice-cold phosphate-buffered saline (PBS) to remove any floating cells and subsequently scraped separately into 50 ml tubes containing 5 ml of PBS on ice. Both the planktonic and the sessile bacterial suspensions were centrifuged at 8,000 g for 10 min at 4°C and the bacterial pellet washed with ice-cold PBS; this procedure was repeated for six times. After being centrifuged for the last time, dry bacterial pellets were prepared by completely removing the PBS. Twenty-four specimens per bacterial strain (2 technical replicates × 3 biological replicates × 2 time points × 2 culture conditions) were prepared and stored at −20°C until analyses.

### Top Down Proteomics – 2D-DIGE

The proteome of three specimens from independent planktonic and sessile cultures of *S. epidermidis* ATCC 35984 and *S. epidermidis* clinical isolate GOI1153754-03-14 were analyzed in duplicate. Cells were suspended in lysis buffer composed of 7 M urea, 2 M thiourea, 30 mM Tris, 4% w/v 3-[(3-cholamidopropyl) dimethylammonio]-1-propanesulfonate hydrate (CHAPS) and 1 mM phenylmethylsulfonyl fluoride (PMSF) and then lysed by means of 0.1 mm zirconium silica beads. All reagents were purchased from Sigma-Aldrich except when otherwise specified.

Briefly, 10 vortex cycles of 60 s were interspersed by 9 min of cooling on ice. After the vortexing cycles, samples were centrifuged at 15,000 g for 10 min at 4°C and the supernatant was collected in new, marked Eppendorf tubes. Protein concentration was determined using the Plus-One 2D-Quant kit (GE Healthcare), according to the manufacturer’s guidelines. Finally, the pH of the extracted protein was adjusted to 8.5–9 by adding a few microliters of 1 M NaOH.

Afterward, protein minimal labeling was performed by mixing 100 μg of samples with 800 pmol of Cy3 (as internal standard) and Cy5 dye (CyDye DIGE Fluor minimal dyes; GE Healthcare), following the manufacturer’s protocol. Before the first separation step, labeled proteins were added to a solution containing rehydration buffer (7 M urea, 2 M thiourea, 2% w/v CHAPS, 65 mM DTT, 0.5% w/v ampholine (IPG buffer pH 3.0–10.0 NL, GE Healthcare), and 1% w/v bromophenol blue. Each biological triplicate was run in duplicate (*n* = 6 gels per condition). The Immobiline DryStrip gels (IPG strip, pH 3-10 NL, 24 cm, GE Healthcare) were actively rehydrated and subsequently separated by isoelectric focusing (IEF) in an Ettan IPGphor electrophoresis unit (GE Healthcare) gradually increasing the voltage as follows: 30 V (6 h), 60 V (6 h), 200 V (1 h), 500 V (1 h), 1,000 V (1 h), 2,000 V (1 h), 3,000 V (1 h), ramping from 3,000 to 8,000 V in 4 h and finally, 8,000 V until the cumulative voltage reached 70 kVh. After IEF, each strip was reduced for 15 min in 4 ml of a solution containing 6 M urea, 375 mM Tris–HCl buffer (pH 8.8), 2% w/v SDS, 20% v/v glycerol and 65 mM DTT. Then, strips were alkylated in 4 ml of the same solution with 135 mM iodoacetamide (IAA) instead of DTT.

The IPG strips were then loaded on 12% w/v acrylamide gels and the second dimension separation was conducted using the Ettan DALT II Electrophoresis Unit (GE Healthcare). In the first separation step, 2 mA per gel was applied for 2 h, then 5 mA per gel for 1 h and finally 15 mA per gel until the complete migration of the bromophenol blue front line.

### Top-Down Proteomics – Image Acquisition and Analysis

A total of 24 bidimensional maps per bacterial strain were scanned by means of Ettan DIGE Imager (GE Healthcare) and images were subjected to data analysis using the DeCyder 2D 7.0 software (GE Healthcare). The gels were first analyzed using a differential in-gel analysis (DIA) module to normalize the Cy3 and Cy5 signals of each gel. The DIA data sets were then submitted to the biological variation analysis (BVA) module, to match and calculate the average of signal abundances for each protein spot across the 24 gels. The statistical analyses were performed using the DeCyder Extended Data Analysis (EDA) module. For each experimental group, spots present in at least 80% of the samples were considered. To identify statistical differences in the expression of proteins between experimental groups, Student’s *t*-test was performed at a significance level *p* < 0.05 preceded by normality verification (Shapiro-Wilk test). In case the *t*-test was not applicable, the non-parametric Mann-Whitney test was used. False discovery rate (FDR) was applied to minimize the error rate. Along with the univariate analysis, principal component analysis (PCA) was performed to support the biological interpretation of the obtained results. The statistical analyses were performed using the DeCyder Extended Data Analysis (EDA) module. Relationships among spot maps were visualized according to the intensity values of protein spots exhibiting statistically significant changes in abundance. All spot maps were distributed in a two-dimensional space, along with the first two principal components, PC1 and PC2 that represented the largest sources of variation in the experimental data set.

### Top-Down Proteomics – Protein Identification

Protein identification was performed by MALDI-TOF mass spectrometry (MS). Briefly, a new set of gels loaded with 400 μg of unlabeled proteins was run following the same electrophoretic protocol described for the 2D-DIGE technique. After the second separation step, gels were stained with a total-protein fluorescent dye (Krypton, Thermo Fisher Scientific) and images of the bidimensional maps acquired with a Typhoon 9200 laser scanner (GE Healthcare). Spot, indicated as variated by statistical analysis, were picked from the gels by means of Ettan spot picker robotic system (GE Healthcare). After the destaining with a solution 50% methanol/50 mM ammonium bicarbonate (AMBIC, Sigma-Aldrich), the excised spots were incubated with 30 μl of 6 ng/μl trypsin (Promega) in 10 mM AMBIC solution overnight at 37°C. Then, 1 μl of retrieved peptides was spotted with an equal volume of 10 mg/ml α-cyano-4-hydroxycinnamic acid matrix (α-CHCA) dissolved in 70% acetonitrile/30% water/0.1% trifluoroacetic acid spotted on a steel target plate and processed by an Ultraflex III MALDI-TOF/TOF mass spectrometer (Bruker-Daltonics; Billerica, MA, United States) in positive reflectron mode. For external calibration, the Peptide Mix calibration mixture was used (Bruker-Daltonics: m/z: 1,046.5418, 1,296.6848, 1,347.7354, 1,619.8223, 2,093.0862, 2,465.1983, 3,147.4710). A total of 1000 laser shots was taken per spectrum. To select monoisotopic peptide masses, mass spectra were analyzed with FlexAnalysis 3.3 software (Bruker-Daltonics). Peak lists were analyzed by MASCOT version 2.4.1 algorithm against NCBIprot_20180429 (Taxonomy Firmicutes) using BioTools v. 3.2 (Bruker Daltonics). For the database search, the parameters carbamidomethylation of cysteines and oxidation on methionines were set as fixed and variable modifications, respectively; one missed cleavage site per peptide was allowed, and maximal tolerance was established at 25 ppm. The significant threshold was set at *p* < 0.05. To confirm the identification obtained, MS/MS spectra were acquired in LIFT mode with 4–8 × 10^3^ laser shots using the instrument calibration file. For fragmentation, precursor ions were manually selected and the precursor mass window was automatically set. The following parameters were used for the database search: carbamidomethylation of cysteines and oxidation on methionine were set for fixed and variable modifications, respectively, maximum of one missed cleavage per peptide was established, and the mass tolerance was set to 30 ppm for precursor ions and to a maximum of 0.5 Da for fragments. The confidence interval for protein identification was set to 95% (*p* < 0.05), and only peptides with an individual ion score above the identity threshold were considered correctly identified.

### Bottom-Up Proteomics – Peptide Sample Preparation

The shotgun mass spectrometry analysis was performed on *ad hoc* bacterial pellets of both sessile and planktonic *S. epidermidis* GOI1153754-03-14 collected and stored as previously reported. Briefly, cell pellets were lysed with 10 cycles of 60 s vortexing interposed by 9 min cooling on ice using a lysis buffer composed of 100 mM Tris-HCl pH 7.6, 8 M urea and 1 mM PMSF. Subsequently, samples were centrifuged 10,000 g for 10 min at 4°C and the supernatant collected in new, marked Eppendorf tubes. The protein concentration in the collected supernatant was assayed by means of the Plus-One 2D-Quant kit (GE Healthcare) and 100 μg of extracted proteins were transferred in a pre-conditioned 30 K MWCO spin column devices (Millipore). Proteins were reduced with 50 μl of 100 mM DTT in 50 mM AMBIC for 30 min at 40°C. After that, the alkylation step was carried out by adding 50 μl of a 55 mM IAA solution and incubating the samples at RT in the dark for 30 min. A wash step was performed after reduction and alkylation steps using 200 μl of 50 mM AMBIC by centrifugation at 10,000 g for 15 min. Furthermore, four additional washes were performed to clear any remaining of Tris-HCl, DTT, IAA, or urea. Finally, 5 μl of sequencing grade trypsin (0.4 μg/μl, Promega) were added to 50 μl of the protein samples and incubated at 37°C for 16–18 h. Later, spin columns were centrifuged at 10,000 g for 15 min to collect tryptic peptides and a final wash with 50 μl of 50 mM AMBIC was performed.

### Bottom-Up Proteomics – LC-MS/MS

Peptide samples were reconstituted in HPLC buffer A (0.1% formic acid) and separated on a Dionex UltiMate 3000 HPLC System with an Easy Spray PepMap RSLC C18 column (250 mm, internal diameter of 75 μm) (Thermo Fisher Scientific), adopting a five steps ACN/formic acid gradient (5% ACN in 0.1% formic acid for 5 min, 5–35% ACN in 0.1% formic acid for 139 min, 35–60% ACN in 0.1% formic for 40 min, 60–100% ACN for 1 min, 100% ACN for 10 min, at a flow rate of 0.3 μl/min), and electrosprayed into an Orbitrap Tribrid Fusion (Thermo Fisher Scientific, Bremen, Germany). The LTQ-Orbitrap was operated in positive mode in data-dependent acquisition mode to automatically alternate between a full scan (350–2,000 m/z) in the Orbitrap (at resolution 60,000, AGC target 1,000,000) and subsequent CID MS/MS in the linear ion trap of the 20 most intense peaks from full scan (normalized collision energy of 35%, 10 ms activation). Isolation window: 3 Da, unassigned charge states: rejected, charge state 1: rejected, charge states 2+, 3+, 4+: not rejected; dynamic exclusion enabled (60 s, exclusion list size: 200). Mass spectra were analyzed using MaxQuant software (version 1.6.3.3). The initial maximum allowed mass deviation was set to 6 ppm for monoisotopic precursor ions and 0.5 Da for MS/MS peaks. Enzyme specificity was set to trypsin/P, and a maximum of two missed cleavages were allowed. Carbamidomethylation was set as a fixed modification, while N-terminal acetylation and methionine oxidation were set as variable modifications. The spectra were searched by the Andromeda search engine against the *S. epidermidis* Uniprot sequence database (2,492 proteins, release 1 December 2019). Protein identification required at least one unique or razor peptide per protein group. Quantification in MaxQuant was performed using the built in XIC-based label free quantification algorithm using fast LFQ. The required FDR was set to 1% at the peptide, 1% at the protein and 1% at the site-modification level, and the minimum required peptide length was set to seven amino acids.

### Bottom-Up Proteomics – Bioinformatics

The ClueGo (v. 2.5.6) and CluePedia (v. 1.5.4) plugins of the software Cytoscape (v. 3.7.2.) were used for functional enrichment and cluster analysis of the over or under expressed proteins. Proteins were classified according to Kyoto Encyclopedia of Gene and Genomes (KEGG) and by gene ontology (GO) annotations in biological process, cellular compartment and molecular function.

Redundant terms were grouped according to the kappa score of 0.4 and only pathways with a *p* < 0.05 were taken into account (Bindea et al., 2009). All the aforementioned analysis were performed based on the *S. epidermidis* ATCC 35984 (RP62A) annotations. To overcome the lack of complete annotation, a manual survey of data was contextually performed. Statistical analysis of proteins identified by LFQ was carried out by means of Perseus software (v. 1.4.0.6) (Tyanova et al., 2016).

Proteins considered to be significantly regulated were further analyzed by STRING v. 11.0^1^ to establish potential interactions and functional relationships at the highest confidence level (0.900).

## Results

### Comparative Proteomic Analysis by 2D-DIGE

**FIGURE 1:**
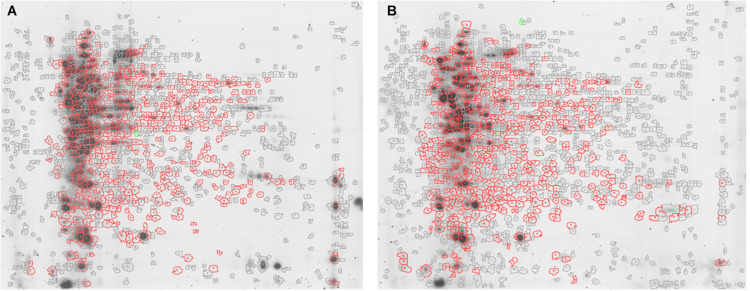
Representative 2D-DIGE maps. Gels show **(A)** *S. epidermidis* ATCC 35984 and **(B)** *S. epidermidis* GOI1153754-03-14 proteomic profile after 48 h of culture. In particular, the proteins differentially expressed in the two culture conditions are circled in red.

**FIGURE 2:**
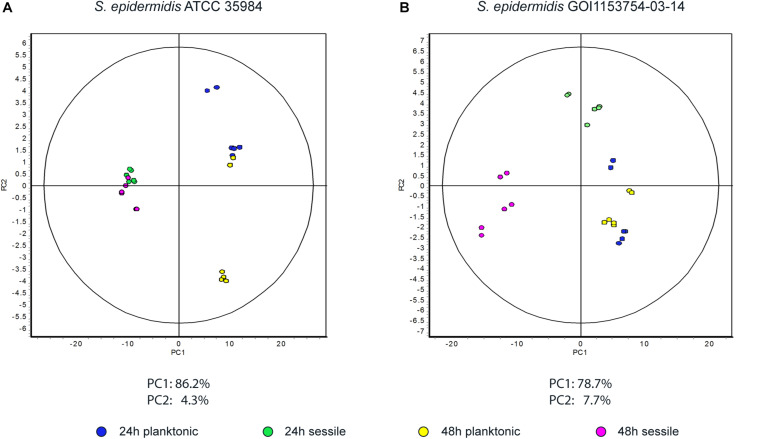
Principal component analysis. Graphs represent **(A)** the spot distribution in *S. epidermidis* ATCC 35984 and **(B)** the spot distribution of *S. epidermidis* GOI1153754-03-14, according to the culture conditions and time points reported in the color legend.

Approximately 575 ± 16 protein spots were detected in each gel. The 2D-DIGE comparative proteomic analysis highlighted a transformed proteomic profile of both *S. epidermidis* reference strain ATCC 35984 and clinical isolate GOI1153754-03-14 after 48 h of culture (Figure 1). An overview of the spot distribution according to the experimental groups was determined by PCA and is reported in Figure 2.

*S. epidermidis* ATCC 35984 is known to have a faster growth rate compared to the clinical isolate strain (Bottagisio et al., 2019); this is characterized by a different proteomic profile even after 24 h of either sessile or planktonic culture (data not shown). Since the aforementioned culture conditions did not make it possible to determine which proteins are crucial for the initial steps of biofilm formation process, attention was then focused only on the restricted number of proteins differentially expressed by *S. epidermidis* GOI1153754-03-14 sessile and planktonic forms at the beginning of the biofilm formation process after 24 h of culture.

Statistical analysis determined that 55 proteins over 558 spots were differentially expressed in *S. epidermidis* as a result of diverse culture conditions (i.e., shake flask and DFR) after 24 h. Of the 55 proteins, 38 were identified by MS. The entire list of identified proteins is reported in Table 1 along with the *p*-values resulting from the statistical comparison between sessile and planktonic *S. epidermidis* clinical isolates after a 24-h culture.

**TABLE 1:** List of significant protein identified by 2D-DIGE between planktonic and sessile *S. epidermidis* GOI1153754-03-14 after 24 h of culture.

UniProt ID	Accession number	Protein name	Sessile VS planktonic (*p*-value)
TIG_STAEQ	Q5HNM8	Trigger factor	↑ (0.00220)
TPIS_STAEQ	Q5HQV2	Triose-phosphate isomerase	↑ (0.00083)
TPIS_STAEQ	Q5HQV2	Triose-phosphate isomerase	↑ (0.00138)
TPIS_STAEQ	Q5HQV2	Triose-phosphate isomerase partial	↓ (0.00917)
PTG3C_STAEQ	Q5HL73	PTS system glucose-specific EIICBA component	↓ (0.00535)
ALF1_STAEQ	Q5HL21	Fructose-bisphosphate aldolase class 1	↓ (0.00231)
LDH_STAEQ	Q5HL31	L-lactate dehydrogenase	↓ (0.0032)
MDH_STAEQ	Q5HR46	Malate dehydrogenase	↓ (0.000989)
Q5HNL0_STAEQ	Q5HNL0	Citrate synthase	↓ (0.0016)
Q5HQ26_STAEQ	Q5HQ26	Succinate dehydrogenase, flavoprotein subunit	↓ (0.000326)
ACNA_STAEQ	Q5HPJ0	Aconitate hydratase A	↓ (0.00408)
GLMS_STAEQ	Q5HM69	Glutamine–fructose-6-phosphate aminotransferase	↓ (0.00898)
GLMS_STAEQ	Q5HM69	Glutamine–fructose-6-phosphate aminotransferase	↓ (0.0000149)
GLMS_STAEQ	Q5HM69	Glutamine–fructose-6-phosphate aminotransferase	↓ (0.00177)
NAGB_STAEQ	Q5HRH8	Glucosamine-6-phosphate deaminase	↓ (0.00333)
ALD1_STAEQ	Q5HMA0	Aldehyde dehydrogenase SERP1729	↓ (0.00172)
A0A2G7HXB3_STAEP	A0A2G7HXB3	NADP-dependent oxidoreductase	↓ (0.0000188)
Q5HL33_STAEQ	Q5HL33	Alpha-acetolactate decarboxylase	↓ (0.000513)
BUTA_STAEQ	Q5HKG6	Diacetyl reductase [(S)-acetoin forming]	↓ (0.00847)
Q5HP34_STAEQ	Q5HP34	Dihydrolipoyl dehydrogenase	↓ (0.0000481)
Y1888_STAEQ	Q5HLU4	Putative 2-hydroxyacid dehydrogenase	↓ (0.0000504)
EFG_STAEQ	Q5HRK5	Elongation factor G	↑ (0.00483)
CODY_STAEQ	Q5HPT7	GTP-sensing transcriptional pleiotropic repressor CodY	↓ (0.00739)
GLYA_STAEQ	Q5HMB0	Serine hydroxymethyltransferase	↓ (0.000284)
A0A4Y7VS80_STAEP	A0A4Y7VS80	FAA hydrolase family protein	↓ (0.0029)
FTN_STAEQ	Q5HN41	Bacterial non-heme ferritin	↓ (0.00265)
ASP23_STAEQ	Q5HM47	Alkaline shock protein 23	↓ (0.0000504)
ASP23_STAEQ	Q5HM47	Alkaline shock protein 23	↓ (0.00675)
ASP23_STAEQ	Q5HM47	Alkaline shock protein 23	↓ (0.0000188)
ASP23_STAEQ	Q5HM47	Alkaline shock protein 23	↓ (0.00000807)
Y1273_STAEQ	Q5HNJ5	Putative universal stress protein SERP1273	↓ (0.0000733)
A0A0N1EHR1_STAEP	A0A0N1EHR1	DNA starvation/stationary phase protection protein	↓ (0.000162)
Q5HR68_STAEQ	Q5HR68	Flavohemoprotein, putative	↓ (0.000681)
TPX_STAEQ	Q5HNJ2	Thiol peroxidase	↓ (0.00144)
TPX_STAEQ	Q5HNJ2	Thiol peroxidase	↓ (0.00486)
SODM_STAEQ	Q5HNZ5	Superoxide dismutase (Mn/Fe)	↓ (0.0000193)
SODM_STAEQ	Q5HNZ5	Superoxide dismutase (Mn/Fe)	↓ (0.00545)

*Arrows indicate up- or down regulated proteins in sessile bacteria when compared to their planktonic counterpart.*

2D-DIGE analysis revealed that the majority of the changes in the proteomic profile of *S. epidermidis* GOI1153754-03-14 occurred when planktonically cultured. Indeed, only 4 of the 38 identified proteins (2 proteoforms of triose-phosphate isomerase, trigger factor, elongation factor G) showed an increased expression in sessile bacteria as the result of biofilm development after a 24-h culture. Of the four aforementioned up regulated proteins, the enzyme triose-phosphate isomerase (tpi, Q5HRC9/SERP0264) was identified. This housekeeping enzyme was significantly up regulated in the sessile clinical isolate when compared to its planktonic counterpart (*p* < 0.001). Among differently expressed proteins, elongation factor G (EF-G, Q5HRK5/SERP0188) was found to be over expressed after 24-h of biofilm formation (*p* < 0.01). Finally, the expression of chaperon protein trigger factor (TF, Q5HNM8/SERP1239) was higher in sessile than in planktonic *S. epidermidis* GOI1153754-03-14 (*p* < 0.01).

Differently, the remaining 34 identified proteins were found to be down regulated by sessile *S. epidermidis* GOI1153754-03-14 when dynamically cultured in DFR for 24 h (Table 1).

In terms of metabolic pathways, these results indicate that sessile bacteria have a reduced metabolic rate compared to their planktonic counterpart in which proteins linked to the tricarboxylic acid (TCA) cycle (i.e., aconitate hydratase, succinate dehydrogenase, citrate synthase, malate dehydrogenase) and glycolysis/gluconeogenesis (e.g., lactate dehydrogenase, fructose-bisphosphate aldolase, PTS system glucose-specific EIICBA component, etc.) were over expressed.

Furthermore, an increase in stress response was found in bacteria grown in the planktonic form compared to sessile microorganisms. Indeed, among the differentially expressed proteins, the putative universal stress protein (asp_23) was identified. Besides that, almost 29% of the over expressed proteins of planktonic clinical isolates were related to the response to stressful conditions.

### Label Free Quantification

LFQ analysis was performed on sessile and planktonic *S. epidermidis* GOI1153754-03-14 after a 24-h culture.

Label free LC-MS/MS analysis identified 1112 proteins within the samples, and among them, 494 were shown to be statistically up regulated and 366 down regulated in sessile *S. epidermidis* GOI1153754-03-14 when compared to planktonic cells.

**FIGURE 3:**
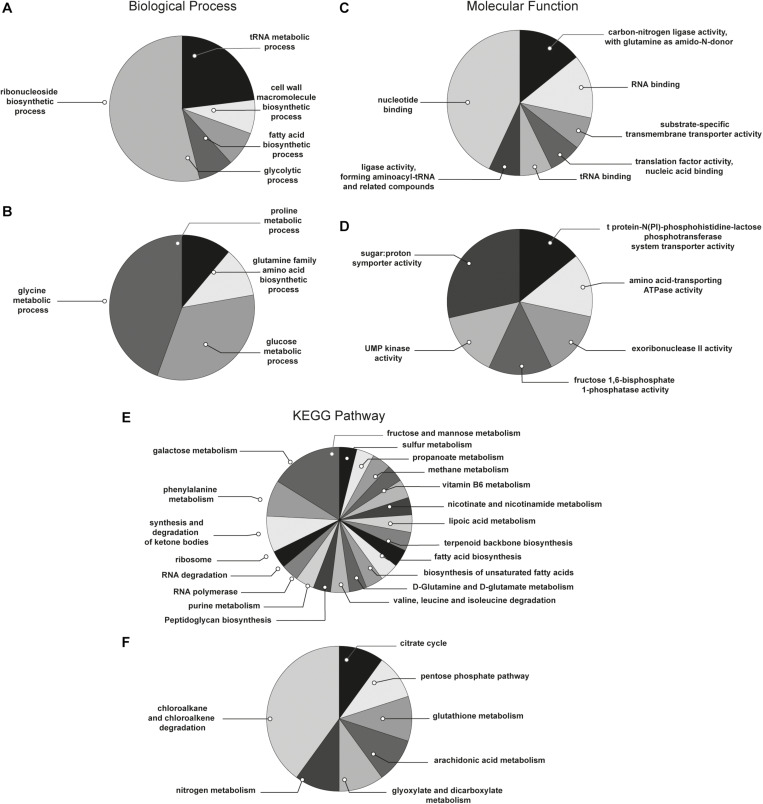
Functional enrichment and cluster analysis. The pie chart reports the most represented biological processes of **(A)** sessile and **(B)** planktonic *S. epidermidis* GOI1153754-03-14; the molecular functions of **(C)** sessile and **(D)** planktonic *S. epidermidis* GOI1153754-03-14 and the KEGG pathways of **(E)** sessile and **(F)** planktonic *S. epidermidis* GOI1153754-03-14.

**FIGURE 4:**
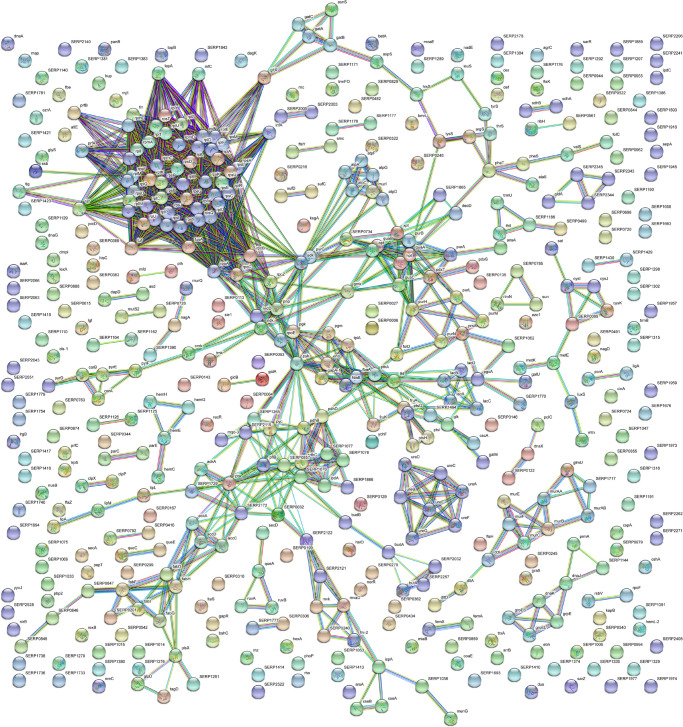
STRING cluster analysis. The network represents the interactions of differentially expressed proteins in sessile *S. epidermidis* GOI1153754-03-14 at the highest confidence level (0.900) after 24 h of culture.

**FIGURE 5:**
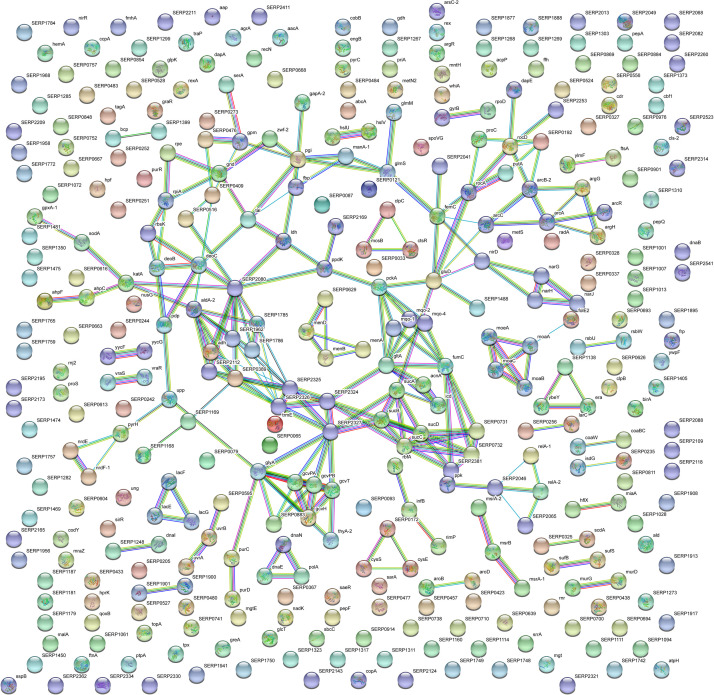
STRING cluster analysis. The network represents the interactions of differentially expressed proteins in planktonic *S. epidermidis* GOI1153754-03-14 at the highest confidence level (0.900) after 24 h of culture.

With the aim to obtain a more detailed picture of the function of identified proteins according to their experimental group, analyses of differentially expressed proteins were performed by means of ClueGO and CluePedia plugins of the software Cytoscape. In particular, enrichment analysis of GO categories for biological processes and molecular functions (Figures 3A–D, respectively) and analysis using the KEGG pathways (Figures 3E,F) were performed to categorize the identified proteins differentially expressed by the sessile and planktonic *S. epidermidis* clinical isolate. Furthermore, cluster analysis using STRING software was also performed to further delineate the interaction of differentially expressed proteins in sessile (Figure 4) and planktonic (Figure 5) *S. epidermidis* at the highest confidence level (0.900).

According to the comparative analysis of the differentially expressed proteins, activation of the ribonucleoside biosynthetic process in biofilm-forming bacteria occurred after 24 h of culture (Figure 3A). In particular, of the 47 ribosomal proteins identified, 3 were not significantly up regulated in sessile culture indicating an active translation process during the biofilm development (Supplementary File 1). Figure 4 shows a cluster of tightly connected proteins that indicate the activation of ribosomal activity. As expected, the 24-h dynamic culture resulted in the up regulation of proteins involved in the initial attachment and the consequent production of cell wall macromolecules in the sessile clinical isolate. In particular, the over expression of surface-associated proteins such as bifunctional autolysin (Alt, Q5HQB9/SERP0636), extracellular binding protein (Ebh, Q5HPA2/SERP1011), D-alanine-D-alanyl carrier protein ligase (DltA, Q5HQN0/SERP0518) was detected in biofilm-forming *S. epidermidis* clinical isolates (Supplementary File 1). Furthermore the up regulation of peptidoglycan biosynthesis, together with the enhanced metabolism of D-glutamine and D-glutamate was observed (Figure 3E) by the expression of important proteins such as Mur ubiquitin ligase enzymes (Supplementary File 1). Also, monosaccharide metabolism (e.g., galactose, fructose, and mannose) and the synthesis of fatty acids, terpenoid backbone, and lipoic acid metabolism were significantly up regulated (Figure 3E). Finally, the results obtained by the LFQ confirmed the up regulation of the glycolytic enzyme (tpi) found by 2D-DIGE, further supported by a concomitant over expression of other glycolytic enzymes acting as moonlight proteins, such as glyceraldehyde 3-phosphate dehydrogenase (GAPDH, Q5HQV4/SERP0442), and enolase (Q5HQV0/SERP0446) (Supplementary File 1).

In comparison, analysis of planktonic cells indicated an upregulation of enzyme of the TCA, pentose phosphate pathways (PPP) and glyoxylate and dicarboxylate metabolism all implicated in central metabolism (Figure 3D). Interestingly, one of the end products of PPP is nicotinamide adenine dinucleotide phosphate (NADPH), which is used by cells to prevent oxidative stress in the reduction of glutathione via glutathione peroxidase, confirming the up regulation of stress response also found in planktonic clinical isolates by 2D-DIGE. Furthermore, KEGG analysis showed an important upregulation of nitrogen metabolism and chloroalkene degradation, known to be associated with bacterial tolerance (Figure 3F). Finally, the two component system (TCS) members YycFG and ArlRS involved in biofilm formation were found to be upregulated in the planktonic 24-h culture of *S. epidermidis* GOI1153754-03-14, along with proteins related to bacterial aggregation such as accumulation associated protein (aap, Q5HKE8/SERP2398) (Figure 5 and Supplementary File 1).

## Discussion

The study of the pathogenesis of infectious diseases has provided evidence concerning the behavior of staphylococci depending on the environmental conditions using genetic documentation. However, there is still the need to elucidate the relationship between encoded genetic information and the ultimate virulence mechanism (Vandecasteele et al., 2001). In this scenario, the proteomic approach offers a more comprehensive picture of the infective process by analyzing the final products of gene biosynthetic pathways (Monteiro et al., 2015). To have a wider perspective of the mechanisms regulating *S. epidermidis* behavior according to different growth environments, in the present study the proteomic profiles of both the *S. epidermidis* reference strain ATCC 35894 and the clinical isolates GOI1153754-03-14 were compared in order to delineate changes in protein expression of two different bacterial strains belonging to the same species. However, already after 24-h incubation, the proteome of sessile and planktonic *S. epidermidis* ATCC 35984 was completely different. Similarly, the protein expression of the clinical isolate was completely different between sessile and planktonic bacteria after 48 h of culture. Thus, it was not possible to determine which proteins are crucial for the initial steps of biofilm formation. Considering the faster growth rate of *S. epidermidis* ATCC 35984, further analyses should focus on shorter time points.

For the aforementioned reasons, only the results obtained by the analysis of the proteome of *S. epidermidis* GOI1153754-03-14 after a 24-h planktonic and sessile culture by 2D-DIGE were further evaluated using LFQ. 2D-DIGE is a solid and elegant top-down approach able to provide data regarding differential abundance of intact proteins highlighting at the same time changes in proteoform distribution (Gelfi and Capitanio, 2018). In the present study, the majority of proteins detected by 2D-DIGE were shown to be down regulated in sessile bacteria, indicating that membrane and cell-surface associated proteins were underrepresented in our dataset. This is an expected drawback related to in gel analyses since intact hydrophobic membrane proteins and cell surface-associated proteins with low solubility are poorly resolved by in-gel methods (Curreem et al., 2012). Hence, the combination of two different technologies were used to corroborate 2D-DIGE results. Moreover, due to unavailability of specific antibodies, which hampers the verification of even few results by independent technique, the adoption of two independent proteomic approaches allowed indirect validation of the results making the combination of these two approaches significant.

### Protein Profile of Sessile *S. epidermidis* Clinical Isolates

The main challenge when reproducing *in vitro* conditions representative of a specific clinical setting is to choose and standardize a method able to produce consistent outcomes. In particular, it has been demonstrated how the fluid shear stress plays a pivotal role in the biofilm growth (Weaver et al., 2012). Hence, to assess the sessile behavior of *S. epidermidis* in an orthopedic setting, in the present study both the reference strain and the clinical isolate were dynamically cultured by means of a DFR on titanium coupons. This dynamic-flow system constantly perfuses growth medium on the titanium surface on which bacteria are attached, providing nutrients under the pressure of low shear stress (Moormeier and Bayles, 2014). Adherence is the first critical step in the pathogenesis of foreign-body infections due to coagulase-negative staphylococci. Hence, the surface properties of these bacteria mediating the adherence to prosthetic devices immediately caught the attention of the scientific community. Tojo and colleagues described for the first time in 1988 a polysaccharide fraction pivotal for the adherence of *S. epidermidis*, which also proved to be its capsule (Tojo et al., 1988). This galactose-rich capsular polysaccharide/adhesin (PS/A) has a structure and composition similar to the polysaccharide intercellular adhesin (PIA) sharing the common backbone composed of β (1–6)-linked glucosamine residues (Heilmann et al., 1996; Mack et al., 1996). However, PS/A has a considerably higher molecular weight compared to PIA and, similarly to poly N-acetylglucosamine (PNAG), it is not soluble in aqueous solution with neutral pH (Agarwal et al., 2010). Therefore, it can be speculated that the over expression of proteins related to the monosaccharide metabolism (e.g., galactose, fructose, and mannose) might be linked to the production of PS/A, modulating the attachment of *S. epidermidis* to titanium coupons (Chun et al., 1990).

Furthermore, other important surface-associated proteins involved in the non-covalent attachment of bacteria to biomaterials were found to be over expressed in sessile clinical isolates in the present study. For instance, the bifunctional autolysin (Atl), with its dual function mediates the bacterial attachment and the hydrolysis of cell wall thus allowing the release of eDNA, essential component of the first phase of biofilm formation (Heilmann et al., 1997). Another protein crucial for the initial attachment stage is the extracellular matrix-binding protein (Ebh), which is involved in the pathogenesis process favoring the intracellular adhesion as well as the fibronectin binding (Christner et al., 2010). Also D-alanine-D-alanyl carrier protein ligase (DltA) supports bacterial adhesion to implant surfaces by catalyzing the D-alanylation of lipoteichoic acid, modulating the net charge of the cell wall (Agarwal et al., 2010; Brown et al., 2013). Finally, the ATP-dependent Clp protease proteolytic subunit (ClpP) supports bacterial attachment as demonstrated by Wang and colleagues through the generation of a *S. epidermidis* clpP mutants (Wang et al., 2007).

Along with the up regulation of surface-associated proteins, it is noteworthy that the active biosynthesis of peptidoglycan and fatty acids by the sessile *S. epidermidis* clinical isolate was observed. Peptidoglycan is the essential component of the staphylococcal cell wall providing the rigidity and structural support indispensable to withstand the internal osmotic pressure as well as external adverse conditions (Giesbrecht et al., 1998). A group of closely related proteins named Mur ligases are fundamental for the synthesis of peptidoglycan and, therefore, essential for bacterial viability, making them ideal antibacterial targets (Kouidmi et al., 2014). The LFQ of proteins extracted from sessile cells showed an up regulation of the Mur ligases superfamily, except for MurD and MurG, which were over expressed in the planktonic counterpart.

Not only were PS/A and the cell wall actively involved in the adaptation of sessile *S. epidermidis* after 24 h of dynamic culture, but also in the changing of the cytoplasmic membrane as indicated through the up regulation of proteins and enzymes involved in the synthesis of fatty acids, terpenoid backbone, and lipoic acid metabolism. Indeed, the biofilm formation process is associated with a specific physiological behavior of the cytoplasmic membrane, which permeability and fluidity tends to decrease to adapt the harsh environmental conditions (Dubois-Brissonnet et al., 2016).

### Moonlighting Proteins Candidates Expressed by Sessile *S. epidermidis* Clinical Isolates

Both in eukaryotes and prokaryotes, some highly conserved metabolic proteins, chaperones and protein-folding catalysts can perform multiple physiologically relevant roles. In 1999, Jeffery (1999) defined this class of multifunctional proteins as “moonlight proteins.” In particular, some glycolytic enzymes and chaperones can be sometimes associated to the virulence properties of the microorganism (Henderson and Martin, 2011). Indeed, it has been demonstrated that some glycolytic enzymes (i.e., tpi, GAPDH, enolase) act as anchorless moonlight proteins targeting extracellular matrix components (e.g., plasminogen) of the host, being present not only in the microbial cytoplasm but also on the cell surface (Ling et al., 2004; Wu et al., 2008; Furuya and Ikeda, 2011; Mukherjee et al., 2012). In addition, there is also evidence in the literature of intracellular chaperones and elongation factors (i.e., EF-G, EF-Tu) acting as adhesion molecules on the surface of Gram-positive bacteria (Wilkins et al., 2003; Jeffery, 2018). Based on this evidence, it can be speculated that the up regulation of the glycolytic enzymes and elongation factors, from our proteomic integrative analyses, might be related to the initial adhesion and biofilm formation process of the clinical isolate. Elongation factors are part of the superfamily of regulatory GTP hydrolases (G proteins) and, EF-G plays a crucial role in the elongation and ribosome translocation steps of the protein translation process (Koripella et al., 2012). The important role of EF-G in staphylococcal virulence is well-known and characterized to the point that it is the target of the bacteriostatic antibiotic fusidic acid (Godtfredsen et al., 1962). Indeed, fusidic acid inhibits protein synthesis by blocking the turnover of EF-G from the ribosome (Verbist, 1990; Fernandes, 2016). Unfortunately, the bacteriostatic nature of fusidic acid encourages the acquisition of fitness-compensatory mutations of the fusA gene encoding EF-G, leading to an alteration of the antibiotic target and reducing the susceptibility to fusidic acid among staphylococci (McLaws et al., 2011). Finally, the up regulation of TF confirmed the important role of moonlight proteins in the initial steps of the biofilm formation process, in which cells rearrange their physiological process to survive harsh conditions. In particular, TF is the only highly conserved, ribosome-associated chaperone and peptidyl-prolyl cis/trans isomerase characterized in bacterial species (Hoffmann et al., 2010). These intracellular/cell surface proteins are triggered to compensate for environmental changes (e.g., variations in pH, shortage of oxygen or metabolites, changes in metal ion concentration, etc.) and their moonlight activity can be strategic for a better understanding tissue colonization, for the identification of biomarkers of disease progression, and for the development of novel therapeutics and vaccines (Jeffery, 2015, 2018).

### Biofilm-Like Behavior of Planktonic Aggregates

Although planktonic bacteria were grown under vigorous agitation to prevent their adhesion and the subsequent biofilm formation, LFQ analysis revealed the presence of proteins linked to biofilm-like behavior. A previous study on the proteome of *S. epidermidis* GOI1153754-03-14 demonstrated the ability of this clinical isolate to form aggregates after 72 h of culture (Bottagisio et al., 2019). This peculiar behavior may be a survival mechanism to cope with harsh conditions after prolonged culture in a media without renewed nutrients (Bottagisio et al., 2019). In the present study, planktonic *S. epidermidis* showed the ability to aggregate after 24 h and synthesize cell wall molecules which contribute to this phenotype. In particular, the accumulation associated protein (aap)was found to be over expressed in planktonic compared to sessile bacteria. The aap mediates the biofilm formation process in a PIA-independent manner by supporting the cell-to-cell adhesion and aggregation process (Rohde et al., 2005). Staphylococci have evolved many different defense strategies to survive the presence of both exogenous and endogenous oxidants (Gaupp et al., 2012). Accordingly, the NADPH end product of the up regulated PPP might be exploited by *S. epidermidis* aggregates to prevent oxidative stress in the reduction of glutathione via glutathione peroxidase (Q5HPN6/SERP0872), confirming the stress response also found in planktonic clinical isolates. These finding along with the over expression of nitrogen metabolism and chloroalkene degradation are known to be associated with bacterial tolerance, usually common in anaerobic and biofilm conditions (Rojony et al., 2019).

Furthermore, the YycFG TCS plays a key role in the rapid adaptation of *S. epidermidis* to environmental changes due to physical, chemical, and/or biological stresses and it is essential for bacterial viability (Wu et al., 2019). Indeed, the deletion of YycFG TCS would cause bacterial death, hence, the silencing by single structured antisense RNA has been consistently proposed in studies to have a pivotal role for these members of the TCS (Xu et al., 2017; Wu et al., 2019). These studies particularly described how the up regulation of the two members of the TCS YycFG (also reported as WalK/WalR) is essential for the regulation of autolysis, biofilm formation, cell wall metabolism, and cellular aggregation (Qin et al., 2006; Xu et al., 2017). YycFG TCS was shown to be over expressed in the planktonic condition compared to sessile culture. Similarly to YycFG, also ArlRS is a member of the TCS with a key role in the regulation of biofilm formation (Burgui et al., 2018), and it was up regulated in planktonic aggregates. It has been demonstrated that ArlRS modulates biofilm formation in an ica-dependent manner in *S. epidermidis* (Wu et al., 2012). The transcription of *icaADBC* is triggered by ArlRS and then regulated by different factors such as Sigma factor B regulator protein (rsbU, Q5HME8/SERP1680) and staphylococcal accessory regulator A (sarA, Q5HRB9/SERP0274) which increase PIA production and S-ribosylhomocysteine lyase (LuxS, Q5HM88/SERP1741) that negatively influence biofilm formation through the secretion of autoinducer 2 in response to changes in cell density (Wu et al., 2012). Hence, the ica-dependent behavior of planktonic *S. epidermidis* aggregates might be correlated with the results obtained in the present study. Indeed, the expression of rsbU and sarA was significantly higher in cells grown in agitation compared to sessile bacteria in which the ica-dependent pathway was negatively regulated by the over expression of LuxS. Furthermore, it has been reported that the down regulation of sarA leads to the over expression of extracellular matrix binding protein (Ebh, Q5HPA2/SERP1011), acting as an intracellular adhesin and contributing to the biofilm formation process. Similarly, the up regulation of Ebh is tightly linked to the over expression of metalloproteinase (sepA, Q5HKU0/SERP2252) which activates the proteolytic function of bifunctional autolysin (Atl, Q5HQB9/SERP0636) (Christner et al., 2012), which is confirmed by the results obtained in the present study.

## Conclusion

The analysis of the proteome of *S. epidermidis* GOI1153754-03-14 demonstrates the importance of external stimuli to differentiate a biofilm lifestyle behavior. Indeed, not only the presence of biofilm-related proteins was detected in settled cell populations scraped from sandblasted titanium coupons, but also in *S. epidermidis* planktonic aggregates when triggered by vigorous agitation. These findings indicate how signals imposed by micro environmental stress/pressure differently activate pathways to form biofilm.

The standardization of culture conditions by the use of the DFR allowed for reproducible sessile cultures. However, on the other hand, it might constitute the main drawback of the study. Indeed, the proteome of an organism is extremely dynamic and even slight variations in the growth conditions under which the proteome is studied might be reflected in the expression of a panel of proteins. In this context, the study design plays a critical role and, for this important reason, the results were elaborated in the context they belong. Therefore, further studies are essential to support the obtained findings to unravel the complex mechanisms of biofilm formation of this emerging pathogen according to the tested experimental conditions.

The study of the behavior of coagulase-negative staphylococci clinical isolates through proteomics is pivotal for the identification of bacterial hallmarks in orthopedic patients with suspected, subclinical PJI. Further studies will be essential to support these findings to further delineate the complex mechanisms of biofilm formation of *S. epidermidis*. Furthermore, they could provide the groundwork for the identification of specific biomarkers potentially leading to a significant improvement in the diagnosis and correct management of low-grade PJIs, enabling clinicians to receive crucial diagnostic indications before surgical treatment.

## Data Availability Statement

The datasets generated for this study can be found in the Supplementary Material.

## Author Contributions

MB, CG, and AL conceived and designed the study. MB grew the cultures under the assistance and the guidance of EL-P and GJ. MB and AB collected and prepared the microbiological samples. MB, DC, PB, and ET performed the proteomic analysis and analyzed the proteomic data. MB wrote the first draft of the manuscript. MB, CG, DC, AB, and AL provided assistance in the manuscript writing. MB, CG, DC, AB, AL, EL-P, and GJ critically revised the manuscript. EL-P and GJ proofread for English grammar. All authors contributed to the article and approved the submitted version.

## Conflict of Interest

The authors declare that the research was conducted in the absence of any commercial or financial relationships that could be construed as a potential conflict of interest.

## Footnotes

1 https://string-db.org/
