# Supplementary material for: Phenotypic Modulation of Biofilm Formation in a Staphylococcus epidermidis Orthopedic Clinical Isolate Grown Under Different Mechanical Stimuli: Contribution From a Combined Proteomic Study
Source: Front Microbiol. 2020 Sep 8;11:565914. doi: 10.3389/fmicb.2020.565914 (PMC7505995; doi:10.3389/fmicb.2020.565914)
Supplement: Supplementary file 2 [file Table_2.DOCX]

Supplementary Material

# Supplementary Data

Supplementary File 1 contains the complete dataset of 2D-DIGE and LFQ results, along with the lists of proteins referring to the STRING analyses of both sessile and planktonic *S. epidermidis* GOI1153754-03-14.
